# Rooting Ability of *Eucalyptus dunnii* Maiden Mini-Cuttings Is Conditioned by Stock Plant Nighttime Temperature

**DOI:** 10.3390/plants15020335

**Published:** 2026-01-22

**Authors:** Matías Nión, Silvia Ross, Jaime González-Tálice, Leopoldo Torres, Sofía Bottarro, Mariana Sotelo-Silveira, Selene Píriz-Pezzutto, Fábio Antônio Antonelo, Arthur Germano Fett-Neto

**Affiliations:** 1 Departamento de Biología Vegetal, Facultad de Agronomía, Universidad de la República, Av. Eugenio Garzón 809, Montevideo 12900, Uruguay; sross@fagro.edu.uy (S.R.); torresleopoldo655@gmail.com (L.T.); sofiabottarro25@gmail.com (S.B.); msotelo@fagro.edu.uy (M.S.-S.); selepiriz@gmail.com (S.P.-P.); 2 Departamento Forestal, Facultad de Agronomía, Universidad de la República, Av. Eugenio Garzón 780, Montevideo 12900, Uruguay; jgonzalez@fagro.edu.uy; 3 Plant Physiology Laboratory, Center for Biotechnology and Department of Botany, Federal University of Rio Grande do Sul (UFRGS), Porto Alegre 91501-970, Brazil; fabioantonioantonelo@gmail.com

**Keywords:** vegetative propagation, adventitious rooting, stock plants, low night temperature, gene expression

## Abstract

Clonal propagation often must incorporate heaters to warm stock plants and stabilize growth. This study investigates the impact that different temperature regimes for stock plants have on the rooting capacity of mini-cuttings derived therefrom. Experiments were conducted in growth chambers using two clones of *Eucalyptus dunnii* Maiden, with clone A’s rooting being moderately better that that of clone B in commercial production. Root primordia differentiation and elongation were faster in clone A than clone B. Stock plants were maintained for one month under two temperature conditions: Δ0 (26/26 °C day/night) and Δ10 (26/16 °C). The main results indicate that rooting significantly decreased with the reduction in nocturnal temperature. Clone A exhibited a 38% reduction in rooting, whereas clone B showed a more pronounced decrease of 65%. In cold nights, soluble carbohydrates at the cutting bases dropped by approximately 25% considering both clones, and overall foliar nutrients also decreased. Cutting base transcript profiles revealed that cold nights decreased the expression of efflux auxin transporter PIN1, increased expression of auxin catabolism-related enzyme DAO, and that expression of auxin nuclear receptor TIR1 remained stable. Fine management of clonal gardens by adjusting thermal conditions can optimize the physiological status of donor plants and enhance the rooting potential and establishment of the derived cuttings.

## 1. Introduction

Planted forests of eucalypts have become widespread worldwide. The main reasons for this expansion are the capacity of these trees to adapt to diverse environmental conditions and the large array of products they provide [1,2]. Nursery production systems rely heavily on mini clonal gardens for vegetative propagation, which involves growing stock plants that produce shoots for subsequent rooting [3]. *Eucalyptus dunnii* Maiden emerges as a promising forest species for producing high-quality cellulose pulp, while also exhibiting greater tolerance to low temperatures and frosts compared to *Eucalyptus grandis* W. Hill [4]. Cold and frost-tolerant eucalypts like *E. dunnii* are needed to supply pulpwood in subtropical climates, such as Uruguay and southern Brazil, where these environmental factors are common in winter. However, the recalcitrance of this species in developing adventitious roots and the variability of its rooting performance [5,6] represent significant challenges for large-scale propagation and considerably increase production costs [7].

Vegetative propagation relies on adventitious rooting (AR), a developmental process by which new roots are established in zones where roots are not generally present, often deriving from non-pericyclic tissues [8]. It is an ordered process comprising three distinct stages called induction, initiation, and elongation (also referred to as expression). The induction phase involves non-anatomical changes (molecular and biochemical events), initiation begins with the cell divisions that define the new meristem, and elongation comprises the meristem growth to form a root [9,10]. The duration and success of each stage depend not only on the optimization of the rooting environment but also on the proper management of the stock plants, as their physiological status is the starting point for the entire process [11,12]. Following this reasoning, numerous studies have attempted to modulate adventitious rooting by altering environmental conditions such as light quality, light flux, temperature, CO_2_ concentration, and mineral nutrition [13]. The present study is part of a concerted effort to improve clonal propagation of *E. dunnii* for supplying subtropical climate plantations. Herein the focus is on the role of the stock plant growth temperature regime on mini-cutting yield and rooting.

Temperature is among the most frequently controlled environmental factors in nurseries due to its relevant effects. The growth temperature of stock plants can interfere with nutrient uptake [3], regulate the production of cuttings and their rooting percentage [6,14], affect source–sink relationships within shoots [15], and influence auxin homeostasis [16]. Specifically, nighttime temperature plays an important role by affecting processes such as plant respiration, repair of damaged photosystems, and carbohydrate translocation [17,18,19]. The heating of stock plant sand beds can affect shoot production and the subsequent rooting of mini-cuttings in different ways [20]. Nighttime temperature can also impact the following day’s photosynthetic activity via nocturnal carbohydrate accumulation, thereby altering source–sink relationships [15] and the carbohydrate concentration in the stem base. An adequate and steady export of carbohydrates from the source to the sink is a crucial requirement for successful adventitious root formation at the base of stem cuttings [16]. Auxin concentration in the apical leaves can also be modulated by nighttime temperature. High nighttime temperatures accelerate the dark reversion rate of *PhyB-PfrB* to *PhyB-PrB* in leaves, releasing *PHYTOCROME INTERACTING FACTOR 4* (*PIF4*) from inhibition. The latter upregulates genes from the *YUCCA* family involved in auxin biosynthesis [21,22]. Given that carbohydrates and auxins are fundamental regulators of adventitious rooting [16], analyzing metabolites or transcripts associated with these pathways in relation to temperature treatments provides valuable insight into rooting mechanisms.

Applied studies have aimed to overcome rooting constraints by modulating stock plant temperature. Trueman et al. [14] reported that growth temperatures of 33/28 °C (day/night) for stock plants increased both the number of shoots produced and the rooting percentage in *E. dunnii*. Batista et al. [23] found that mini-tunnel-maintained temperatures increased an average of 1.8 °C throughout the year, yielding a 10% increase in the rooting of *E. urophylla* × *E. dunnii* hybrid cuttings during summer months. Despite the recognized importance of temperature in production, there is a scarcity of studies delving into the underlying metabolic and molecular mechanisms altered by this factor [13].

Given that modern propagation systems often incorporate heaters to control nighttime temperature, this study aimed to explore, under growth chamber conditions, the effects of nighttime temperature applied to stock plants on the adventitious rooting capacity of cuttings in two commercial clones of *E. dunnii*. Since AR is considerably determined by carbohydrates, nutrient status, and auxin homeostasis, this study hypothesizes that nighttime temperature modulates one or more of these factors, thereby affecting propagation efficiency. To this end, we investigated the physiological status of the stock plants by evaluating foliar nutrition and carbohydrate concentration. Anatomical changes throughout root development and the expression of genes linked to auxin homeostasis during the induction phase were recorded in the cuttings. The relationship between these variables, budburst capacity, and rooting performance is discussed. It is anticipated that the gathered data may help to design improved scalable strategies for commercial greenhouses.

## 2. Results

Stock plants of two *E. dunnii* clones with different rooting competence were subjected to controlled environmental modulation treatments and monitored for one month. The impacts of each treatment were studied by collecting stock plant branching and mini-cutting rooting data. Anatomical, biochemical, and molecular analysis were performed in the basal 2 cm of the cuttings, where the adventitious rooting process takes place.

### 2.1. Anatomy

**Figure 1:**
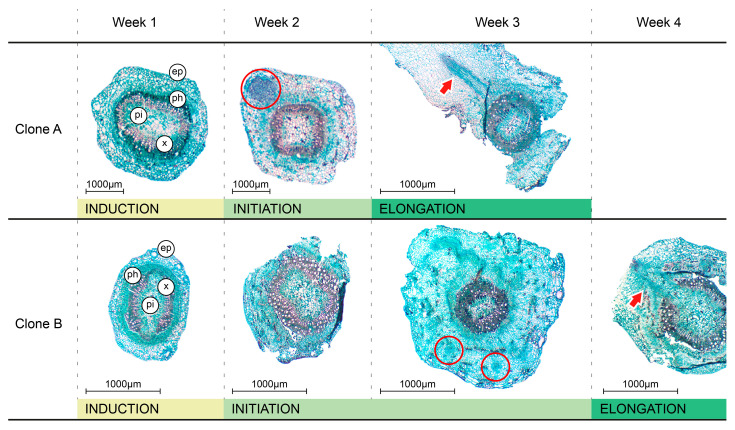
Comparative anatomical changes in the basal portion of *Eucalyptus dunnii* mini-cuttings derived from Clone A and B during different phases of adventitious root development. Representative sections of basal stems 1, 2, 3, and 4 weeks after excision. Five stems were sampled per timepoint and clone. Abbreviations: ep, epidermis; ph, phloem; x, xylem; pi, pith; red circles indicate newly formed root meristems; red arrows indicate emerged roots.

An initial experiment was carried out to delimit the rooting phases and explore differences between the two clones. Anatomical changes of cutting bases obtained from stock plants grown under Δ0 temperature conditions were examined weekly for one month. This period encompassed the time from severance to rooting of mini-cuttings (Figure 1). During the first week, both clones exhibited the regular anatomical distribution of a secondary stem without any visible root meristematic clusters. A single layer of epidermal cells covers several layers of ground tissue, consisting of parenchyma and sclerenchyma. Vascular tissue is composed of the bilateral phloem, typical of Myrtaceae, and the xylem. Except for the vascular tissues, no secondary growth was evident in the other regions of the stem. Subsequently, anatomical differences became apparent between clones. By the second week, clusters of cells with prominent nuclei, indicative of meristematic activity, were frequently observed in the cortex. Unlike Clone B, which displayed several small division points, Clone A exhibited fewer but larger ones. Root elongation was also faster in Clone A. By the third week, cuttings from Clone A showed emerged roots extending through the cortex with clear vascular connections with the original cutting, whereas Clone B still frequently exhibited small primordia. By the fourth week, the cuttings of Clone B also displayed adventitious root development with vascular connections. In both clones, roots originated directly from the vascular cylinder and nearby tissues, although in some mini-cuttings calli were also formed, as shown in the third week in Clone B. Occasional roots that originated from calli were not included in the morphological analyses.

### 2.2. Branching and Rooting

**Figure 2:**
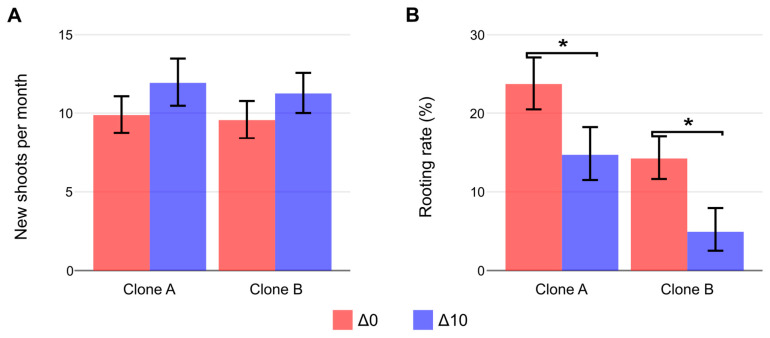
Productivity of stock plants (Clone A and Clone B) under constant (Δ0, 26/26 °C day/night) and reduced nighttime temperature (Δ10, 26/16 °C day/night) after one month of cultivation. (**A**) Monthly number of new shoots produced by stock plants and (**B**) final rooting rate of mini-cuttings obtained (%). Bars represent mean ± SEM. Asterisks (*) denote significant differences between treatments for each clone (orthogonal contrast, *p* < 0.05; n = 18 stock plants for shoot production, n = 3 experimental units of at least 20 mini-cuttings for rooting).

The number of harvestable branches (Figure 2A) was not different between clones or experimental conditions (*p* > 0.05), with a general monthly mean of 11.1 ± 4.6 new sprouts per stock plant. Overall rooting rates were rather low (under 30%). Nonetheless, rooting was higher (*p* > 0.05) in Clone A than Clone B (Figure 2B). Both genotypes showed reduced rooting capacity (*p* > 0.05) when nighttime temperatures were decreased by 10 °C (Δ10), highlighting the key role of this environmental parameter on propagation. Clone A exhibited a 38% reduction in rooting, whereas Clone B showed a more pronounced decrease of 65% (Figure 2B). Mortality was also recorded, and the 5% confidence intervals for Δ0 and Δ10 were [33–44%] and [57–67%], respectively.

### 2.3. Carbohydrates

**Figure 3:**
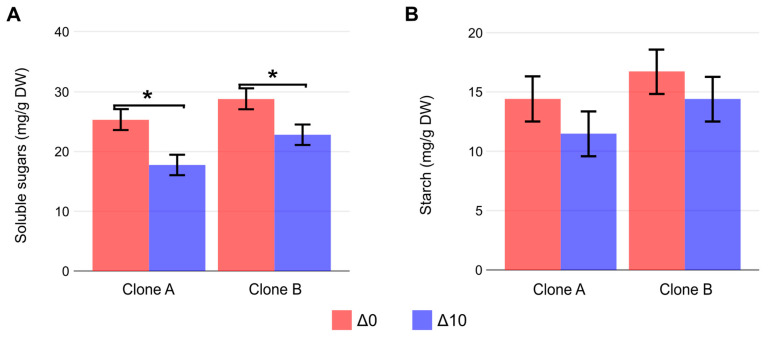
Carbohydrate concentration at the 2 cm basal portion in Clone A and Clone B under constant (Δ0, 26/26 °C day/night) and reduced nighttime temperature (Δ10, 26/16 °C day/night) at mini-cutting excision. (**A**) Soluble sugars (mg/g DW). (**B**) Starch concentration (mg/g DW). Bars represent mean ± SEM (n = 3). Asterisks (*) denote significant differences between treatments for each clone (orthogonal contrast, *p* < 0.05, n = 3 pools of 100 mg; each pool was derived from 3 to 4 mini-cuttings).

To explore differences in carbon allocation at excision, carbohydrates were quantified. Soluble sugars were significantly reduced by the decrease in nighttime temperatures (*p* < 0.001) (Figure 3A). This reduction was more pronounced in Clone A. On the other hand, starch concentration was not significantly affected by treatment or clone type (Figure 3B).

### 2.4. Nutrient Profile

**Figure 4:**
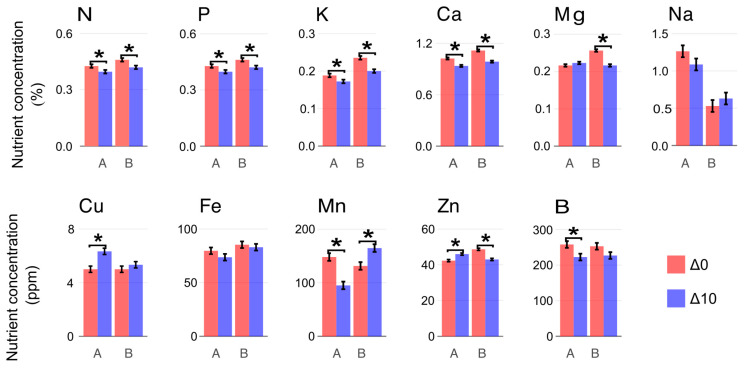
Foliar nutrient profile of mini-cuttings at the time of excision of Clone A and Clone B under constant (Δ0, 26/26 °C day/night) and reduced nighttime temperatures (Δ10, 26/16 °C day/night). Concentrations of N, P, K, Ca, Mg, Na (% DW); Cu, Fe, Mn, Zn, and B (ppm DW). Bars represent mean ± SEM (n = 3). Asterisks (*) denote significant differences between treatments for each clone (orthogonal contrast, *p* < 0.05, n = 3 pools of 100 mg; each pool was derived from 3 to 4 mini-cuttings).

The nutritional profile of the stock plants was significantly affected by exposure to low nighttime temperatures (Figure 4). Despite some exceptions, the overall trend was of foliar nutrient concentration reduction. The nonessential nutrient sodium was not significantly affected by the treatments in either clone. Except for magnesium (Clone A), the leaf concentrations of all measured macronutrients were reduced. In contrast, the micronutrient responses were clone-dependent. Clone A exhibited a reduction in manganese (Mn) and boron (B) concentrations alongside an increase in zinc (Zn) and copper (Cu). Conversely, Clone B showed a decrease in Zn and an increase in Mn quantities.

### 2.5. Gene Expression Analysis

**Figure 5:**
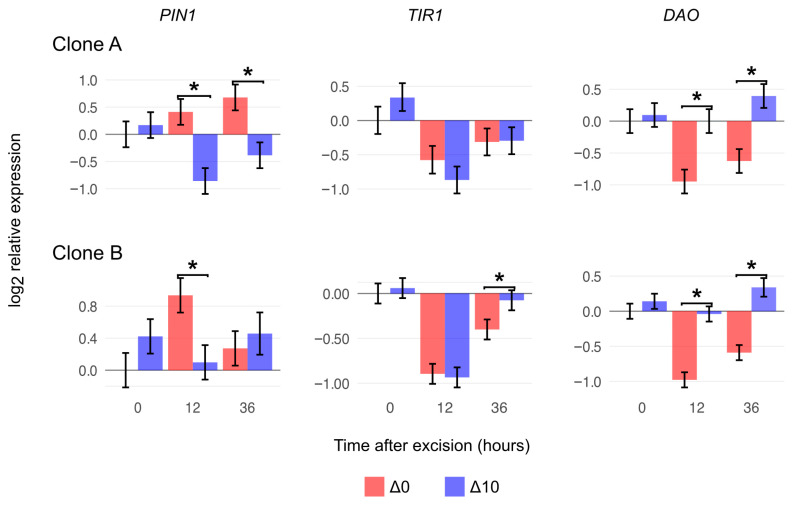
Log2-transformed relative expression profile (at the 2 cm basal portion of mini-cuttings) of *PIN-FORMED 1* (*PIN1*), *TRANSPORT INHIBITOR RESPONSE 1* (*TIR1*), and *DIOXYGENASE FOR AUXIN OXIDATION* (*DAO*) of Clone A and Clone B under constant (Δ0, 26/26 °C day/night) and reduced nighttime temperatures (Δ10, 26/16 °C day/night) at 0, 12, 36 h post excision from the stock plant. Bars represent mean ± SEM (n = 3 pools, 4 mini-cutting bases per pool). Asterisks (*) indicate differences by orthogonal contrast (*p* < 0.05) between treatments at one timepoint.

The expression profiles of key genes involved in the adventitious rooting process were monitored at the base of the cuttings during the early hours of the induction phase (Figure 5). Nighttime temperature affected several gene expression profiles. Unlike *PIN1* and *DAO*, the expression pattern of *TIR1* was significantly affected only by time (*p* < 0.05), with no significant differences between treatments. In both clones, relative expression levels decreased 12 h after excision. *PIN-FORMED 1* expression was significantly affected by the interaction between clone and time. Clone A showed a reduction in relative expression in response to the nighttime temperature drop, whereas Clone B continued to increase its expression, albeit at a low level. *DAO* expression levels were also significantly affected by the interaction between clone and time, displaying similar expression patterns in both clones. In both cases, relative expression was significantly reduced in the Δ0 treatment following excision.

## 3. Discussion

With the aim of generating information for the industry, which demands a deeper understanding of the basis of vegetative propagation, the objective of this work was to investigate how the nocturnal temperature of stock plants determines the rooting potential of the mini-cuttings derived from them. To this end, stock plants were grown under controlled conditions, and the rooting of mini-cuttings obtained from them was investigated. Key components such as carbohydrate concentration and foliar nutrition at mini-cutting harvest, as well as the expression of genes involved in auxin metabolism during the early root induction phase, were analyzed. The results indicate that reduced nighttime temperatures had a negative impact on rooting. The analysis of these components provides insights into the mechanisms underlying this reduction.

### 3.1. Anatomy

The study of anatomical traits provides a valuable tool for identifying and temporally delimiting each phase of AR development, also allowing for the identification of possible physical barriers and other structural limitations to its progression. In the present experiment, anatomical changes were examined on a weekly basis to detect possible differences between clones. The clones appeared to differ in the timing and pattern of root primordia establishment. Notably, Clone B exhibited a longer initiation period without the clear formation of a root meristem, as was observed in Clone A. Also, in Clone B, the appearance of sclerified tissue within the cortical region suggests that an occurrence of cell divisions resembling those observed during callus formation was more frequent. In a comprehensive study, Eliyahu et al. [24] analyzed the anatomy of calli formed in *Eucalyptus* cuttings and reported results consistent with those obtained in the present study. Data suggest that differences in histological patterns may be consistent with the distinct abilities of each clone to generate adventitious roots.

### 3.2. General Productivity

The number of mini-cuttings and their respective rooting percentages are the main components of propagation productivity. Traditionally, only rooting percentage has been considered; however, to obtain the highest possible number of cuttings, branching also plays an important role [6,14]. Rossi and Isabelle [25] evaluated how nighttime temperature modulates sprouting in *Picea maritima* under controlled conditions. Their findings indicated that daytime temperature had a stronger effect than nighttime temperature. Similarly, in the present experiment, plants were maintained under the same diurnal temperature regime, and the sprouting rate was not significantly affected (*p* < 0.05). In contrast, a nursery investigation with *E. dunnii* in southern Brazil using heated stock plant sand beds during colder seasons (triggered when temperatures dropped below 15 °C) recorded significant inhibition of sprouting with no effect on the rooting of derived mini-cuttings relative to unheated control sand beds [20]. In the case of the present study, overall productivity was determined primarily by the rooting rate of cuttings, with treatment effects on rooting being more pronounced than on sprouting. Both clones showed significantly reduced rooting under cold night conditions, with Clone B exhibiting the most pronounced decrease (65%). To further investigate the physiological causes in rooting decline, carbohydrate concentration, foliar nutrient profiles, and the expression of key genes were determined and analyzed.

### 3.3. Carbohydrates

Carbohydrates and auxins are two key components in the formation of adventitious roots [16]. Beyond serving as an energy source and providing carbon skeletons, carbohydrates such as glucose, sucrose, and trehalose also contribute to signaling during this process by interfering with gene expression [26,27]. Therefore, understanding their homeostasis can help to explain specific rooting outcomes. Since carbohydrates in this experiment were quantified in the basal portion and not in the leaves of the cuttings, it is important to consider how translocation processes between the source organs and the base of the cutting might be affected by nighttime temperature. The efficient partitioning of carbohydrates between the new sink of developing roots at the cutting base and the shoot apex could be critical for AR [16].

Soluble sugar amounts at the base of the cuttings were reduced under low nighttime temperatures, while starch content remained unaffected. Nighttime temperature can play an important role by affecting processes such as plant respiration, repair of damaged photosystems, and carbohydrate translocation [17,18]. Low nighttime temperature can indirectly affect photosynthetic activity via carbohydrate accumulation in leaves during the period, influencing source–sink relationships and, consequently, the carbohydrate allocation to the stem base. Domingues-Junior et al. [28] studied how cold exposure (10 °C) for a 24 h period alters the source–sink relationship between leaves and stems in *E. grandis*. Their results indicated that cold reduces stem carbohydrate concentration, as these metabolites accumulate in leaves for the synthesis of protective compounds against cold. In grapevine, a 10 °C decrease in nighttime temperature has been associated with a reduction in net assimilation rate on the following day, possibly due to the accumulation of total carbohydrates in leaves. This increased concentration may contribute to feedback inhibition of photosynthesis. Leaf carbohydrate accumulation in cold conditions is attributed to a low respiratory rate and potential limitations in translocation to other plant organs [15]. Similarly, in a cold-sensitive cucumber cultivar, a 10 °C reduction in nighttime temperature led to the accumulation of sucrose, stachyose, and galactinol in mature leaves, whereas sucrose, glucose, and fructose concentrations in fruits remained unchanged. Analysis of peduncles, where stachyose is catabolized to sucrose, revealed a significant increase in stachyose and a reduction in sucrose, likely associated with inhibited translocation. This inhibition was particularly linked to reduced enzymatic activity, potentially resulting from low ATP concentrations. The observed reduction in cucumber fruit growth rates under cold nights suggests that sink activity is more affected than source supply [29].

The decrease in carbohydrates at the cutting base observed in the present study may be primarily due to impaired translocation due to the lower temperature during the night [28]. A possible contribution of photosynthetic feedback inhibition in leaves cannot be ruled out. The reduction in carbohydrate availability at cutting bases could negatively impact rooting.

### 3.4. Mineral Nutrition

Carbohydrate and hormone metabolism, along with cell division, are directly influenced by mineral nutrients [30], making nutrition a key factor in rooting predisposition [3]. The role of mineral nutrients in adventitious rooting is further highlighted by studies showing that rooting-phase-specific nutrient formulations in aseptic cultures enhance rooting in *Eucalyptus globulus* Labill [31]. Furthermore, since the nutritional status is already established at wounding, the condition of the stock plants critically determines the rooting response [16].

In this study, leaf nutrient status was significantly affected, with almost all macronutrients decreasing in Δ10, and with micronutrients exhibiting clone-specific patterns. These changes may help explain differences in the decline of rooting performance among clones. Although all macro- and micronutrients are essential for plant growth and development, alterations in Fe, Zn, Cu, and Mn concentrations have been reported as critical for adventitious rooting in *Eucalyptus dunnii*. Low Cu and Zn levels have been associated with increased tissue oxidation and poor rooting rates [32]. Both micronutrients are integral components of the enzyme superoxide dismutase (CuZnSOD), which contributes to H_2_O_2_ balance during adventitious rooting. Decreased leaf nutrient availability might affect ROS modulation at excision. This regulation is crucial for lignification processes and for protecting peroxidases from inactivation by superoxide (O_2_^−^) [33,34]. In addition to the impact on reactive oxygen species homeostasis, Zn deficiency disrupts auxin metabolism because this metal is required for the biosynthesis of tryptophan, a precursor of indole-3-acetic acid (IAA) [35,36]. Mn also plays a key role in plant oxidative processes as a component of MnSOD, exerting similar effects to Cu and Zn but with specific mitochondrial activity [37]. Moreover, Mn enhances the activity of enzymes involved in IAA degradation and lignin polymerization, such as peroxidases [38]. Interestingly, a decrease in Mn during the induction phase of *E. globulus* cuttings significantly increased rooting, possibly by favoring auxin regulatory activity at the wound site [31].

In this experiment, Clone A was less affected than Clone B by the low nighttime temperature treatment. The higher levels of Zn and Cu, together with the reduction in Mn, may have contributed to the regulation of oxidative stress in the cuttings while limiting IAA degradation during the induction phase. In contrast, Clone B not only displayed an opposite micronutrient pattern but also showed a reduction in Mg content, which may be associated with the pronounced decrease in rooting capacity observed in Δ10.

### 3.5. Gene Expression

Following excision, cells undergo transcriptional reprogramming during the induction phase to establish the conditions required for the formation of a new root meristem [39,40]. It has been proposed that polar auxin transport (PAT) plays a pivotal role during this period. Several studies of both woody and herbaceous species have shown that early accumulation of indole-3-acetic acid (IAA) in the rooting zone is crucial for cell reprogramming, and that this increase results primarily from active auxin transport rather than from local biosynthesis [10,13]. Within a few hours, both IAA levels and tissue sensitivity markedly decline [10,41]. Previous studies in *Eucalyptus* have reported enhanced transcription of *PIN-FORMED 1*-like genes after excision, associated with improved rooting performance [42]. However, in the present study, twelve hours after excision, cuttings of both clones derived from cold-night-exposed stock plants exhibited reduced *PIN1* transcript levels, suggesting that low temperature interfered with early IAA accumulation. Considering that *DAO* expression increased significantly 12 h after excision in Δ10 for both clones, early auxin accumulation may be reduced due to decreased transport activity combined with enhanced degradation.

*TRANSPORT INHIBITOR RESPONSE 1* (*TIR1*), a nuclear F-box protein belonging to the *TIR1/AFB1–5* receptor family, regulates auxin perception through ubiquitin-mediated signaling [43]. Here we found that the expression of *TIR1* decreased twelve hours after excision, which is consistent with previous observations in *Petunia* [41]. In *E. globulus* microcuttings, a decrease in the expression of *TIR1* was also recorded during root induction in the cambium zone tissues where the first cell divisions leading to root primordia took place [42]. Although under a different sampling scheme and cultivation system (commercial greenhouse sand beds), a study with an *E. dunnii* clone also indicated a decrease in *TIR1* expression during the root induction phase of whole mini-cuttings [6]. Despite this expected transcriptional decrease, no significant differences were detected between temperature treatments, suggesting that stock plant nighttime growth temperature did not interfere with auxin sensitivity based on its nuclear receptor expression. Further investigation is required to clarify the status of other components in auxin responses (e.g., *AUX/IAA* and *ARFs*) and establish the impact of low nighttime temperature on auxin sensitivity.

Based on the time course analyses of *PIN1*, *DAO*, and *TIR1* expressions, the overall results indicate that the growth temperature of stock plants influences auxin status of the cuttings without altering tissue sensitivity to the phytohormone. These findings highlight the importance of donor plant preconditioning to optimize physiological competence for adventitious root development.

## 4. Materials and Methods

### 4.1. Plant Material, Growing Conditions, and Sampling

The experiments were performed with one-year-old stock plants of two *Eucalyptus dunnii* clones that differed in rooting ability. Under commercial greenhouse conditions, the rooting ability of Clone A is approximately 20% higher than that of Clone B, according to information provided by the stock plant supplier (UPM, Paysandú, Uruguay). The plants were established in a commercial greenhouse in 2 L plastic pots filled with composted pine bark. The plants were fertilized daily with 100 mL of a ferti-irrigation solution (S 175 mg/L, B 1.45 mg/L, Ca 162 mg/L, Cu 4.8 mg/L, P 50 mg/L, Fe 6.1 mg/L, Mg 70 mg/L, Mn 2.3 mg/L, NO_3_^−^ 86 mg/L, NH_4_^+^ 57 mg/L, K 150 mg/L, Na 40 mg/L, and Zn 4.5 mg/L).

Eighteen stock plants of each clone were cultivated in controlled-environment growth chambers under the temperature conditions described in Table 1. After pruning, the plants were randomly distributed among the chambers and maintained for one month, after which sprouts were pruned and mini-cuttings were formed. Mini-cuttings were prepared with three to four pairs of leaves (10–12 cm) and placed in plastic tubes pre-filled with the same substrate as used for the stock plants. Since the focus of the present study was on the stock plants, rooting conditions were the same for all experiments (see Table 1, mini-cuttings column). Shoot production data were from 18 stock plants and rooting rates were calculated from triplicates of at least 20 mini-cuttings.

Three replicates of experimental units, each consisting of 50 mini-cuttings representing every combination of clone and temperature treatment were prepared and placed in growth chambers under saturating humidity and bottom heat control, as indicated in Table 1. At excision, triplicate pools of 100 mg of mini-cuttings (three to four per pool) were frozen in liquid nitrogen for carbohydrate quantification and stored at −80 °C. At the same sampling point, three sample groups of leaves from the same number of mini-cuttings were harvested, rinsed with distilled water, and dried at 60 °C for foliar nutrient analysis.

For RNA extraction, three pools of 100 mg of basal segments of mini-cuttings (four per pool) were collected and frozen in liquid nitrogen at 0, 12, and 36 h after excision and stored at −80 °C. Finally, one month after excision, the number of rooted mini-cuttings was recorded to calculate the rooting percentage. Only direct root development patterns were used for quantification (Figure S1).

### 4.2. Anatomical Analysis

In the first experiment, anatomical changes of mini-cutting bases obtained from stock plants grown under control conditions (Δ0 in Table 1) were examined from severance to rooting. Five cuttings were collected weekly throughout one month, fixed in formalin–acetic acid–ethanol (FAA, 5:5:90) for 48 h, and then stored in 70% ethanol. Samples were processed using the paraffin embedding technique [44]. Serial transverse sections (5–10 µm thick) were obtained using a semi-automatic microtome. Sections were stained using the safranin-fast green double-staining method [45]. Observations were carried out under a Nikon E100 light microscope (Tokyo, Japan), and image capture and processing were performed using an Amscope digital camera and Amscope software (version macOS 2.1.18573.20210304; Irvine, CA, USA).

### 4.3. Soluble Sugar Quantification

The extraction of soluble sugars was carried out according to the methods of DuBois et al. [46] and Ruedell et al. [13], with minor modifications. Frozen samples of about 30 mg of fresh weight (FW) were homogenized in liquid nitrogen, extracted with 750 μL of 80% ethanol (*v*/*v*), and incubated in a water bath at 75 °C for 15 min. The extracts were centrifuged at 13,000× *g* for 15 min and the supernatant was recovered. The pellets were re-extracted with 750 μL of 80% ethanol (*v*/*v*). The quantification of soluble sugars was performed according to the method of McCready et al. [47], with minor modifications. One hundred μL of the extract was mixed with 600 μL of freshly prepared anthrone reagent (1 g of anthrone in 500 mL of 72% sulfuric acid (*v*/*v*)). The resulting solution was mixed and kept in a boiling water bath for 11 min. After cooling, the absorbance at 630 nm was determined in a Jasco V-730 spectrophotometer (Tokyo, Japan). The standard curve was established with D-glucose.

### 4.4. Starch Quantification

To quantify starch, pellets obtained from the soluble sugar extraction were used [47]. Pellets were resuspended in 250 μL of distilled water and 320 μL of 52% perchloric acid (*v*/*v*), submitted to sonication in a water bath for 15 min, and centrifuged at 13,000× *g* for 15 min. Extraction was performed twice. Quantification of starch was as described above for soluble sugars. The standard curve was established with D-glucose in 29.2% perchloric acid (*v*/*v*).

### 4.5. Leaf Mineral Status

Fresh cutting biomass from both clones was collected at excision and sent to Laboratorio de Suelos y Aguas (Facultad de Agronomía, Udelar) for leaf nutrient determination. Plant biomass was dried at 60 °C until constant weight and ground to a powder with particle sizes smaller than 1 mm. The powder was incinerated at 500 °C in a muffle furnace. One gram of ash samples was diluted with hydrochloric acid and used to determine the concentrations of phosphorus (P) by the Ascorbic Acid colorimetric method [48]; boron (B) by the Azomethine-H colorimetric method [49]; magnesium (Mg), calcium (Ca), iron (Fe), zinc (Zn), copper (Cu), and manganese (Mn) by Atomic Absorption; and total potassium (K) by Atomic Emission spectrometry [50]. For the Atomic Emission and Absorption determinations, a PerkinElmer PinAAcle™ 500 spectrometer (Waltham, MA, USA) was used.

### 4.6. Gene Expression Analysis

Total RNA was extracted from the basal 20 mm of cuttings using the RNeasy Plant Mini Kit (Qiagen, Hilden, Germany) and treated with RNase-free DNase I (Thermo Scientific™, Waltham, MA, USA). RNA quality was verified by electrophoresis on a 1% agarose gel, and concentration was measured using a NanoDrop Lite spectrophotometer (Thermo Scientific). First-strand cDNA synthesis was performed using 500 ng of total RNA with the RevertAid First Strand cDNA Synthesis Kit (Thermo Scientific™) and oligo (dT) primers. The resulting cDNA was diluted 1:50 in nuclease-free water prior to use.

Quantitative PCR (qPCR) reactions were conducted on a QuantStudio™ Real-Time PCR System, 96-well, 0.1 mL (Applied Biosystems™, Waltham, MA, USA), using the Maxima SYBR Green/ROX qPCR Master Mix (2X) (Thermo Scientific™) in a final volume of 10 µL. Each sample was analyzed with three biological and four technical replicates. Primers for the target genes (Table 2), as well as for the reference genes, were synthesized by Macrogen (Seoul, South Korea) based on the sequences published by Vilasboa et al. [6].

Reference gene selection was based on the assessment of expression stability among commonly used candidates for AR in *E. globulus*, including *SAND-DOMAIN PROTEIN* (*SAND*), *ACTIN2* (*ACT2*), and *HISTONE 2B* (*H2B*) [51]. Stability was evaluated using the NormFinder algorithm of the R package NormqPCR (Version 1.48.0; Kaysville, UT, USA) [52], and *H2B* and *ACT2* were selected as the most stable reference genes. PCR efficiency values (Table S1) were calculated from raw fluorescence data using the qPCR package [53]. Melting temperatures are also listed in Table S1. Representative melting curves (time of excision) are depicted in Figure S2. Relative gene expression levels were determined using the comparative Ct method (ΔΔCt) [54], and the results are presented as log_2_ fold changes.

### 4.7. Statistical Analyses

The experiment followed a 2 × 2 factorial design, with two genotypes (Clone A and Clone B) and two temperature regimes (Δ0 and Δ10). A two-way ANOVA was conducted to evaluate the effects of treatment, genotype, and their interaction. The results of the analysis of variance for each variable are provided in Tables S2–S5.

Data normality was assessed using the Shapiro–Wilk test, and homogeneity of variances was verified using Levene’s test. Arcsine and inverse square root transformations were applied to normalize variables with a binomial distribution. Statistical analyses were carried out in R using the ‘stats’ package [55]. Since no major restrictions affected randomization, orthogonal contrasts were performed to assess the treatment effects within each genotype, with the 1smeans package [56].

## 5. Conclusions

Stock plant growing temperature strongly influenced the rooting capacity of the derived cuttings. Variations in daytime–nighttime temperature regimes significantly affected rooting performance. The results suggest that key processes such as carbohydrate accumulation in stems, foliar nutrient status, and auxin homeostasis-related transcriptional activity at the cutting base are negatively impacted by low nighttime temperatures. This leads to reduced leaf mineral concentrations, lower stem carbohydrate reserves as an energy source, and possibly decreased auxin pools during the induction phase. These findings provide a better understanding of the benefits of temperature control in commercial greenhouses. As a general guideline, it would be advisable to avoid nighttime temperatures far below 20 °C. It is also possible to consider tailoring temperature schemes to specific clones of high interest as a strategy to improve propagation. Further studies are required to validate these effects under operational production conditions.

## Figures and Tables

**Table 1:** Environmental conditions for stock plants and cuttings in growth chambers.

	Stock Plant	Mini-Cuttings
Photoperiod (day/night)	12/12	12/12
Humidity (%)	62.4 ± 10.9	92.7 ± 5.6
Chamber temperature (day/night °C)	Δ0: 25.2 ± 2.2/25.4 ± 1.0 Δ10: 24.8 ± 3.0/16.7 ± 1.7	21.3 ± 1.6
Bottom heat (°C)	OFF	ON at 22.2 ± 0.2
Light flux quality (µmol m^−2^ s^−1^)	340—Full Spectrum	100—Full Spectrum

**Table 2:** Primer sequences for target *PIN-FORMED 1* (*PIN1*), *DIOXYGENASE FOR AUXIN OXIDATION* (*DAO*), and *TRANSPORT INHIBITOR RESPONSE 1* (*TIR1*), and reference genes *HISTONE H2B* (*H2B*) and *ACTIN 2* (*ACT2*).

Gene Symbol	Forward Primer (5′–3′)	Reverse Primer (5′–3′)
*H2B*	CGTCTCAGAAGGGACCAAGG	ACGGGTAACACACAACTTCC
*ACT2*	GCACCGCCAGAGAGGAAATA	CGACTTTGTTGGATTTAGAAGCAC
*PIN1*	TTTGCCCATAACGCTCGTCT	CACCATCCCACCATGTCCAA
*DAO*	AGTGATGGACAAGTCGGGTT	GCTGCCTTCTTTGGACTCAAC
*TIR1*	GTGGCACTATGGTGTGGTGA	AGCTCAGCCAAGTGCAAT

## Data Availability

The original contributions presented in the study are included in the article/Supplementary Materials, and further inquiries can be directed to the corresponding authors.

## Supplementary Materials

The following supporting information can be downloaded at: https://www.mdpi.com/article/10.3390/plants15020335/s1, Figure S1: One-month-old rooted mini-cutting derived from constant temperature conditions (Δ0, 26/26 °C day/night), showing a detail of the basal portion. The image highlights the callus formed at the wound zone and the clear pattern of direct root development immediately above it; Figure S2: Representative post-run melting curves of reference (*HISTONE-H2B*, and *ACTIN 2—ACT2*) and target genes (*PIN-FORMED 1—PIN1*, *DIOXYGENASE FOR AUXIN OXIDATION—DAO*, and *TRANSPORT INHIBITOR RESPONSE 1—TIR1*) corresponding to the time of cutting excision; Table S1: Primer amplification efficiencies and melting curve temperatures for the genes *PIN-FORMED 1* (*PIN1*), *DIOXYGENASE FOR AUXIN OXIDATION* (*DAO*), *TRANSPORT INHIBITOR RESPONSE 1* (*TIR1*), *HISTONE H2B* (*H2B*), and *ACTIN 2* (*ACT2*). Values represent mean ± SD calculated from raw fluorescence data; Table S2: ANOVA significance values of the sources of variation and their interaction regarding the general productivity of stock plants of Clone A and Clone B under constant (Δ0, 26/26 °C day/night) and reduced nighttime temperature (Δ10, 26/16 °C day/night) treatments. Nr of Shoots: Monthly number of new shoots produced. Rooting: Percentage of mini-cutting rooting after one month of the excision. Number of replicates: 18 stock plants for shoot production; n = 3 experimental units with at least 20 mini-cuttings each for rooting. Values refer to Figure 2; Table S3: ANOVA significance values of the sources of variation and their interaction with carbohydrate concentrations at the 2 cm basal portions of mini-cuttings in Clone A and Clone B under constant (Δ0, 26/26 °C day/night) and reduced nighttime temperatures (Δ10, 26/16 °C day/night). Data on soluble sugars (mg/g DW) and starch concentrations (mg/g DW) refer to mini-cutting samples taken right after excision. Number of replicates: n = 3 pools of 100 mg of mini-cuttings (3 to 4 per pool). Values refer to Figure 3; Table S4: ANOVA significance values of the sources of variation and their interaction with the foliar nutrient profile of excised mini-cuttings of Clone A and Clone B under constant (Δ0, 26/26 °C day/night) and reduced nighttime temperatures (Δ10, 26/16 °C day/night). Concentrations of N, P, K, Ca, Mg, Na (% DW); Cu, Fe, Mn, Zn, and B (ppm DW) refer to the leaves of mini-cuttings right after excision. Number of replicates: n = 3 pools of 100 mg, each pool derived from 3 to 4 mini-cuttings. Values refer to Figure 4; Table S5: ANOVA significance values of the sources of variation and their interaction with Log2-transformed relative expression profiles at the 2 cm mini-cutting basal portion of *PIN-FORMED 1* (*PIN1*), *TRANSPORT INHIBITOR RESPONSE 1* (*TIR1*), and *DIOXYGENASE FOR AUXIN OXIDATION* (*DAO*) of Clone A and Clone B under constant (Δ0, 26/26 °C day/night) and reduced nighttime temperatures (Δ10, 26/16 °C day/night) at 0, 12, 36 h post excision from stock plants. Number of replicates: n = 3 pools of 100 mg, each pool derived from 4 mini-cuttings. Values refer to Figure 5.
