# Supplementary material for: Rooting Ability of Eucalyptus dunnii Maiden Mini-Cuttings Is Conditioned by Stock Plant Nighttime Temperature
Source: Plants (Basel). 2026 Jan 22;15(2):335. doi: 10.3390/plants15020335 (PMC12844827; doi:10.3390/plants15020335)
Supplement: Supplementary file 1 [file plants-15-00335-s001.zip › plants-4078909-supplementary.pdf]

Supplementary Material

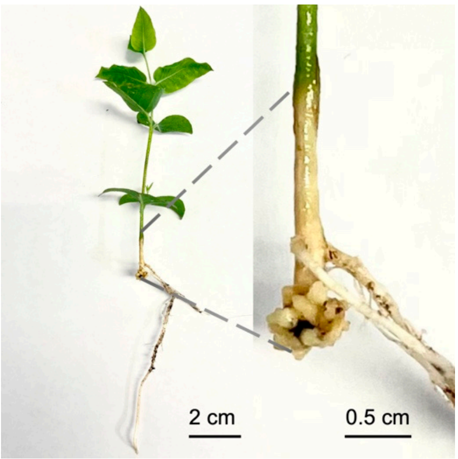

Figure S1. One-month-old rooted minicutting derived from constant temperature conditions (Δ0, 26/26°C day/night), showing a detail of the basal portion. The image highlights the callus formed at the wound zone and the clear pattern of direct root development immediately above it.

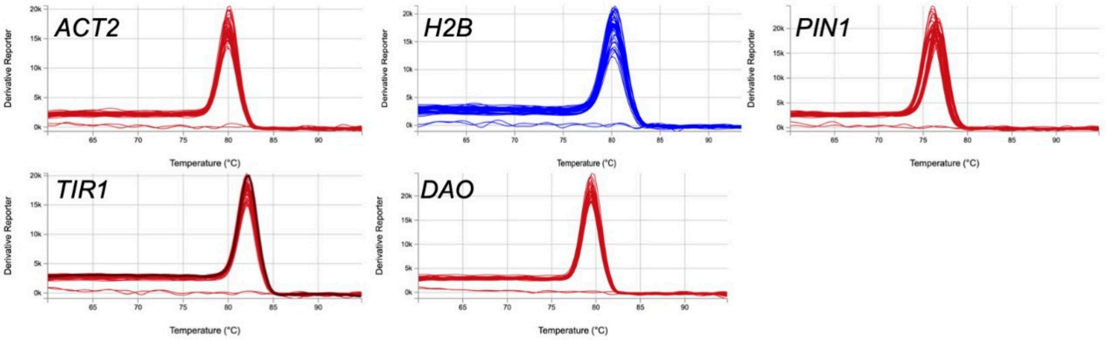

Figure S2. Representative pos-run melting curves of reference (*HISTONE- H2B*, and *ACTIN 2 - ACT2*) and target genes (*PIN-FORMED 1 - PIN1, DIOXYGENASE FOR AUXIN OXIDATION - DAO, TRANSPORT INHIBITOR RESPONSE 1 - TIR1*) corresponding to the time of cutting excision.

Table S1. Primer amplification efficiencies and melting curve temperatures for the genes *PIN-FORMED 1* (*PIN1*), *DIOXYGENASE FOR AUXIN OXIDATION* (*DAO*), *TRANSPORT INHIBITOR RESPONSE 1* (*TIR1*), *HISTONE H2B* (*H2B*), and *ACTIN 2* (*ACT2*). Values represent mean ± SD calculated from raw fluorescence data.

	Amplification Efficiency (%)	MeltCurve Tm (°C)
ACT2	100.10 ± 1.26	80.07 ± 0.78
H2B	99.04 ± 1.09	80.33 ± 0.13
PIN1	94.30 ± 3.84	76.51 ± 0.31
TIR1	98.20 ± 0.92	79.62 ± 0.17
DAO	98.67 ± 0.81	81.29 ± 1.13

Table S2. ANOVA significance values of the sources of variation and their interaction regarding general productivity of stock plants of Clone A and Clone B under constant (Δ0, 26/26°C day/night) and reduced night temperature (Δ10, 26/16°C day/night) treatments. Nr of Shoots: monthly number of new shoots produced. Rooting: percentage of minicutting rooting after one month of the excision. Number of replicates: 18 stock plants for shoot production, n = 3 experimental units with at least 20 minicuttings each for rooting. Values refer to Figure 2.

	Nr of Shoots	Rooting
Genotype	0.798	0.006
Treatment	0.151	0.011
Interaction	0.903	0.576

Table S3. ANOVA significance values of the sources of variation and their interaction of carbohydrate concentrations at the 2 cm basal portions of minicuttings in Clone A and Clone B under constant (Δ0, 26/26°C day/night) and reduced night temperature (Δ10, 26/16°C day/night). Data of soluble sugars (mg/g DW) and starch concentrations (mg/g DW) refer to minicutting samples taken right after excision. Number of replicates, n = 3 pools of 100 mg of minicuttings (3 to 4 per pool). Values refer to Figure 3.

	Soluble Sugars	Starch
Genotype	0.037	0.206
Treatment	0.004	0.205
Interaction	0.658	0.878

Table S4. ANOVA significance values of the sources of variation and their interaction of foliar nutrient profile of excised minicuttings of Clone A and Clone B under constant (Δ0, 26/26°C day/night) and reduced night temperature (Δ10, 26/16°C day/night). Concentrations of N, P, K, Ca, Mg, Na (% DW); Cu, Fe, Mn, Zn, B (ppm DW) refer to leaves of minicuttings right after excision. Number of replicates: n = 3 pools of 100 mg, each pool derived from 3 to 4 minicuttings. Values refer to Figure 4.

	N	P	K	Ca	Mg	Na	Cu	Fe	Mn	Zn	B
Genotype	0.9187	0.017	5.9 e-5	0.00034	0.00105	7.0 e-5	0.066	0.0417	0.0059	0.0283	0.9728
Treatment	0.0009	0.006	0.0008	1.8 e-5	0.00105	0.64	0.007	0.2152	0.2047	0.1475	0.012
Interaction	0.8651	0.610	0.0820	0.1090	0.000113	0.12	0.066	0.5701	0.0003	7.04 e-5	0.6354

Table S5. ANOVA significance values of the sources of variation and their interaction of Log2-transformed relative expression profile at the 2 cm minicutting basal portion of *PIN-FORMED 1* (*PIN1*), *TRANSPORT INHIBITOR RESPONSE 1* (*TIR1*) and *DIOXYGENASE FOR AUXIN OXIDATION* (*DAO*) of Clone A and Clone B under constant (Δ0, 26/26°C day/night) and reduced night temperature (Δ10, 26/16°C day/night) at 0, 12, 36 hours post excision from stock plant. Number of replicates: n = 3 pools of 100 mg, each pool derived from 4 minicuttings. Values refer to Figure 5.

	Clone A			Clone B		
	PIN1	*TIR1*	*DAO*	*PIN1*	*TIR1*	*DAO*
Time	0.2866	0.0025	0.0449	0.3963	6.3e-6	0.0009
Treatment	0.0029	0.8974	0.0007	0.6108	0.2305	1.9e-5
Interaction	0.0215	0.3175	0.0499	0.0326	0.2780	0.0055
